
JOURNAL INFORMATION
==============================
NLM Title Abbreviation: ScientificWorldJournal
Journal ID: ScientificWorldJournal
NLM Journal ID: 101131163
Journal ID: TSWJ

The Scientific World Journal

ISSN: 2356-6140
EISSN: 1537-744X
Publisher: Wiley

ARTICLE INFORMATION
==============================
PMCID: PMC6084634
DOI: 10.1100/tsw.2001.447
Article ID: 850874
Article version: 1
Subjects: Extended Abstract

The Roles of Amylin in the Periphery

Wookey P. J.
Cooper M. E.
Department of Medicine, University of Melbourne, Austin & Repatriation Medical Centre, Repatriation Campus, Heidelberg West, Victoria 3081, Australia
Publication date: 2001
Electronic publication date: 2001 Nov 21
Volume: 1
First page: 23
Last page: 23
Copyright: Copyright © 2001 P. J. Wookey and M. E. Cooper.
Copyright year: 2001
License: This is an open access article distributed under the Creative Commons Attribution License, which permits unrestricted use, distribution, and reproduction in any medium, provided the original work is properly cited.
License URL: https://creativecommons.org/licenses/by/3.0/

==============================
4th International Meeting on Calcitonin Gene-Related Peptide (CGRP)
*TheScientificWorld* (2001) 1(S1), 23
ISSN 1532-2246; DOI 10.1100/tsw.2001.447

### THE ROLES OF AMYLIN IN THE PERIPHERY

P.J. Wookey and M.E. Cooper
Department of Medicine, University of Melbourne, Austin & Repatriation Medical Centre, Repatriation Campus, Heidelberg West, Victoria 3081. Australia

The primary site of amylin biosynthesis lies in the pancreatic islet β-cells, but secondary sites are found in the gastrointestinal tract, dorsal root ganglia, and in the developing kidney where expression appears tightly controlled. The known actions of amylin as a hormone are equally diverse with physiological effects in the CNS in terms of satiety and memory, and in the PNS in gastric emptying and nociception. Furthermore in the periphery amylin has important functions in proximal tubules of the kidney where as a hormone it stimulates sodium/water resorption[1] and is potent as a proliferation factor both during development[2] and regeneration of adult cells[1]. To explain its renal actions amylin binding sites have been identified on the proximal tubules, as well as possible intracellular structures, amylin immunoreactive vesicles. These binding sites on proximal tubules are distinct from the expression of calcitonin receptors and RAMP 3 that have been defined by immunohistochemical staining on other tubules. The actions of amylin in the renal cortex are thought to be important in the maintenance of the polarity of proximal epithelial cells. These functions are considered important in the pathogenesis of hypertension[3] and may be linked to renal complications associated with diabetes. Furthermore in the pancreatic islets amylin may play a role in islet enlargement, an important issue in the progression towards overt diabetes. Recent studies by our group with the amylin gene-deletion mouse model provide further evidence for these roles of amylin in renal development and islet enlargement.

### REFERENCES

1. Harris, P.J., Cooper, M.E., Hiranyachattada, S., Berka, J.L., Kelly, D.J., Nobes, M., and Wookey, P.J. (1997) Amylin stimulates proximal tubular sodium transport and cell proliferation in the rat kidney. Am. J. Physiol. 272, F13–F21.
2. Wookey, P.J., Tikellis, C., Nobes, M., Casley, D., Cooper, M.E., and Darby, I.A. (1998) Amylin as a growth factor during foetal and postnatal development of the rat kidney. Kidney Int. 53, 25–30.
3. Wookey, P.J., Cao, Z., van Geenen, R.C.I., Voskuil, M., Darby, I.A., Komers, R., and Cooper, M.E. (1997) Increased density of renal amylin binding sites in experimental hypertension. Hypertension 30, 455–460.
